# Skeletal Muscle Hyperemia: A Potential Bridge Between Post-exercise Hypotension and Glucose Regulation

**DOI:** 10.3389/fphys.2021.821919

**Published:** 2022-01-31

**Authors:** Thomas K. Pellinger, Chi-An W. Emhoff

**Affiliations:** ^1^Department of Physical Therapy, University of Maryland Eastern Shore, Princess Anne, MD, United States; ^2^Department of Kinesiology, Saint Mary’s College of California, Moraga, CA, United States

**Keywords:** glucose delivery, blood pressure, vasodilation, recovery, type 2 diabetes, histamine receptors

## Abstract

For both healthy individuals and patients with type 2 diabetes (T2D), the hemodynamic response to regular physical activity is important for regulating blood glucose, protecting vascular function, and reducing the risk of cardiovascular disease. In addition to these benefits of regular physical activity, evidence suggests even a single bout of dynamic exercise promotes increased insulin-mediated glucose uptake and insulin sensitivity during the acute recovery period. Importantly, post-exercise hypotension (PEH), which is defined as a sustained reduction in arterial pressure following a single bout of exercise, appears to be blunted in those with T2D compared to their non-diabetic counterparts. In this short review, we describe research that suggests the sustained post-exercise vasodilation often observed in PEH may sub-serve glycemic regulation following exercise in both healthy individuals and those with T2D. Furthermore, we discuss the interplay of enhanced perfusion, both macrovascular and microvascular, and glucose flux following exercise. Finally, we propose future research directions to enhance our understanding of the relationship between post-exercise hemodynamics and glucose regulation in healthy individuals and in those with T2D.

## Introduction

As with healthy individuals, in patients with type 2 diabetes (T2D), regular physical activity offers numerous benefits, including improved blood glucose regulation (reduced HbA1c) (Boulé et al., 2001; Umpierre et al., 2011) increased insulin sensitivity (Winnick et al., 2008), improved clinical symptoms (Wojtaszewski and Richter, 2006) and the delay or prevention of developing cardiovascular disease (Balducci et al., 2012). In addition to these benefits of regular exercise, evidence suggests even one bout of dynamic exercise stimulates increased insulin-mediated glucose uptake (Usui et al., 1998; Oguri et al., 2009) and insulin sensitivity (Devlin et al., 1987; Perseghin et al., 1996; Bordenave et al., 2008) in patients with T2D during post-exercise recovery. These findings highlight the importance of the post-exercise period in optimizing glycemic regulation in this population. In this mini review, we provide a brief overview of the phenomenon known as post-exercise hypotension (PEH) and how the sustained vasodilation that is frequently associated with PEH may affect glucose regulation in both healthy individuals and in people with T2D. In doing so, we focus on the significance of post-exercise blood flow, both macrovascular and microvascular, in the delivery, transport and metabolism of glucose. Finally, we suggest future research directions to advance our understanding of how post-exercise hemodynamics affect glucose regulation in both healthy individuals and those with T2D.

## Overview of Post-Exercise Hypotension

During recovery from an acute bout of dynamic exercise, humans experience PEH, which features a sustained reduction in arterial pressure compared to pre-exercise control levels (Kenney and Seals, 1993). In most circumstances, PEH is associated with post-exercise skeletal muscle vasodilation that is not completely off-set by a still elevated cardiac output (Halliwill et al., 2013). However, occasionally (e.g., in some hypertensive subjects) it has corresponded with a decreased cardiac output accompanied by an increase in systemic vascular resistance (Brito et al., 2014). Numerous mechanisms appear to be responsible for PEH and the sustained post-exercise vasodilation often observed in the previously active skeletal muscle vascular beds. The baroreflex is reset to defend a lower pressure following exercise (Halliwill et al., 1996), which is sometimes associated with a decrease in post-exercise muscle sympathetic outflow (Halliwill et al., 1996) and/or an increase in parasympathetic activity concomitant to the PEH (Park et al., 2006). However, other investigations have found no changes in autonomic modulation (Park et al., 2008; Anunciação et al., 2016) or have observed increased sympathetic activity (Teixeira et al., 2011; Cunha et al., 2016) post-exercise, which suggests that variations in autonomic control may reflect a physiological response to compensate for the fall in blood pressure via the downward resetting of the arterial baroreflex following exercise (Farinatti et al., 2021). For a given level of sympathetic nerve activity, reduced vascular resistance has been observed in the previously active skeletal muscle vascular beds after exercise (Halliwill et al., 2003). Importantly, post-exercise skeletal muscle vasodilation is facilitated locally by activation of both the histamine H_1_ and H_2_ receptors, as combined H_1_ and H_2_-receptor antagonism attenuates PEH by ∼65% and post-exercise vasodilation by ∼80% following 60 min of moderate-intensity dynamic exercise (Halliwill et al., 2013).

PEH has been observed in men and women (Senitko et al., 2002) and in both sedentary and endurance-trained individuals (Senitko et al., 2002; Lockwood et al., 2005; McCord et al., 2006; McCord and Halliwill, 2006). Numerous modes of aerobic exercise may evoke PEH (MacDonald, 2002) and although most investigations have employed large muscle dynamic exercise (e.g., cycle ergometer), PEH and post-exercise vasodilation have also been induced by single-leg dynamic knee extension exercise (Barrett-O’Keefe et al., 2013). In normotensive and hypertensive individuals, in both laboratory and ambulatory studies, PEH has been provoked by varying doses of exercise (MacDonald et al., 2000; Pescatello et al., 2004; Eicher et al., 2010), although the magnitude and duration of PEH are somewhat dose-dependent with regards to exercise intensity (Forjaz et al., 2004; Smelker et al., 2004) and duration (Forjaz et al., 1998; Mach et al., 2005).

## Post-Exercise Glucose Regulation in Healthy Individuals

Skeletal muscle glucose uptake is dependent on several determinants that can be sequentially categorized into either glucose delivery, transport, or metabolism (Wasserman and Ayala, 2005). Delivery of glucose to the interstitial space is determined by arterial glucose concentration, skeletal muscle blood flow, capillary perfusion, and endothelial permeability (Jensen and Richter, 2012). Depending on acute (e.g., physical activity level) or chronic conditions (e.g., disease), any of these steps can be rate-limiting.

During the first 90 min post-exercise, skeletal muscle glucose uptake is enhanced in an insulin-independent manner (Richter et al., 2001; Henriksen, 2002). This elevated muscle glucose uptake corresponds with the peak glycogen synthesis rate in the previously exercised skeletal muscle (Price et al., 1994; Casey et al., 2000), thereby promoting post-exercise glycogen repletion.

Research on both rodents (Schultz et al., 1977; Hespel et al., 1995) and humans (Hickner et al., 1991; Baron et al., 1994; Durham et al., 2003) suggest augmented limb blood flow promotes skeletal muscle glucose uptake, even independent of the vasodilatory influence of insulin. In examining the influence of exercise on this relationship, Hamrin et al. (2011) observed, via skeletal muscle microdialysis, that increased tissue perfusion was associated with enhanced glucose uptake 12 h after the completion of a 2-h bout of moderate-intensity one-legged cycling. Notably, this response was independent of enhancement of insulin responses, as they found similar increases in skeletal muscle glucose uptake in the post-exercising and post-resting legs in response to a hyperinsulinemic-euglycemic clamp.

### Role of Histamine Receptor-Mediated Vasodilation

Several studies employing histamine-receptor blockade have lent support to the notion that sustained skeletal muscle vasodilation following exercise aids in the movement of glucose from the central circulation to skeletal muscles. Pellinger et al. (2010) utilized skeletal muscle microdialysis following 60 min of moderate-intensity cycling exercise to demonstrate that glucose delivery to previously active skeletal muscle is supported by post-exercise vasodilation, as interstitial glucose concentration was reduced when post-exercise hyperemia was blunted by local H_1_- and H_2_-receptor blockade. Subsequently, Emhoff et al. (2011) found that oral H_1_- and H_2_-receptor antagonism reduced both femoral vascular conductance and leg glucose delivery after 60 min of cycling exercise. Interestingly, due to high interindividual variability, skeletal muscle glucose uptake was not universally decreased by the histamine receptor blockade in this study. However, they noted that histamine receptor blockade blunted glucose uptake in subjects who obtained higher absolute oxygen consumptions, suggesting a potential histaminergic impact on glucose uptake and glycogen repletion in individuals who exercise at high workloads. Furthermore, Pellinger et al. (2013) observed that oral H_1_- and H_2_-receptor antagonism reduced whole body insulin sensitivity by 25% in healthy individuals following 60 min of moderate-intensity cycling exercise. This finding suggests that if histaminergic skeletal muscle vasodilation is blunted, so too is delivery of glucose and insulin to skeletal muscle cells, likely attenuating insulin-mediated vasodilation and capillary recruitment. Under these circumstances, greater secretion of insulin would be required in response to a sustained elevation of blood glucose (Pellinger et al., 2013). Taken together, these investigations suggest the importance of histamine-receptor-mediated elevations in skeletal muscle blood flow in glucose regulation following exercise.

### Post-exercise Microvascular Perfusion and Membrane Permeability

To determine the impact of prior acute exercise on insulin-mediated skeletal muscle microvascular blood flow, Sjøberg et al. (2017) employed a euglycemic-hyperinsulinemic clamp 4 h after single-legged exercise by young, healthy males. They found microvascular perfusion was greater 4 h post-exercise and increased 40% more in the previously exercised leg than in the rested leg, in response to insulin stimulation. Furthermore, after insulin stimulation, leg glucose uptake increased 50% more in the previously exercised leg than in the rested leg. Importantly, arterial infusion of the nitric oxide synthase (NOS) inhibitor (L-NMMA) reversed the insulin-induced rise in arterial and microvascular blood flow in both legs and abolished the greater glucose uptake found in the previously exercised leg. Interestingly, the previously exercised muscle had higher insulin signaling at the level of the protein coding gene TBC1D4 (which mediates both exercise and insulin-stimulated GLUT4 translocation) and glycogen synthase activity and this was largely unaffected by L-NMMA. These findings indicate that acute exercise increases skeletal muscle insulin sensitivity via coordinated increases in both microvascular perfusion and insulin signaling (Sjøberg et al., 2017).

Recently, Parker et al. (2021) provided further evidence of elevated microvascular perfusion well after the cessation of dynamic exercise. Utilizing a randomized cross-over design, they investigated the effect of 60 min of moderate-intensity cycling exercise on postprandial skeletal muscle microvascular blood flow responses to a high-glucose mixed nutrient meal consumed 3 h post-exercise. The exercise bout enhanced both femoral artery and muscle microvascular blood flow for up to at least 3 h post-exercise. Moreover, although the high-glucose meal evoked microvascular impairments in each condition, muscle microvascular blood flow remained almost twice that of the non-exercise control condition during the 2 h following the meal consumed post-exercise. These findings are promising regarding the impact of dynamic exercise on individuals with microvascular dysfunction or impairments in glucose regulation.

**FIGURE 1:**
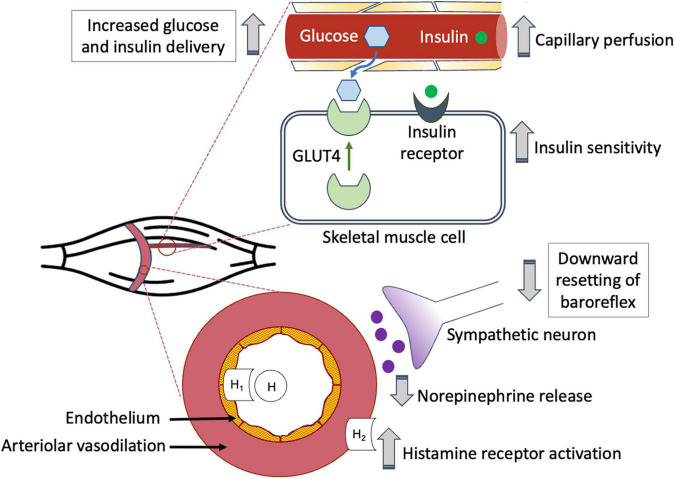
Post-exercise hyperemia facilitates glucose delivery to previously active skeletal muscle.

Both exercise (Kennedy et al., 1999; Flores-Opazo et al., 2020) and insulin-induced (Ryder et al., 2000; Koistinen et al., 2003) GLUT4 translocation to the skeletal muscle membrane promote glucose uptake. However, research indicates that increases in insulin-stimulated GLUT4 translocation are significantly less than the increases in glucose transport (Thorell et al., 1999). In an effort to clarify this discrepancy, McConell et al. (2020) used measurements of leg glucose uptake and skeletal muscle interstitial glucose concentrations to estimate insulin-stimulated muscle membrane permeability in healthy young men 4 h after performing 60 min of 1-legged knee-extensor exercise during a submaximal euglycemic-hyperinsulinemic clamp. Using this novel technique, they found that during insulin stimulation, muscle membrane permeability to glucose and glucose uptake increased roughly twice as much in the previously exercised leg than in the rested leg. In addition, although muscle membrane permeability to glucose did not change in either leg with ATP (an endothelium-dependent vasodilator) infusion, this caused both leg blood flow and glucose uptake to rise substantially, with the greater increases found in the previously exercised leg. These findings reinforce the possible role of increased post-exercise blood flow to support sufficient glucose uptake during recovery from exercise. Figure 1 provides an overview of the relationship between PEH, post-exercise blood flow, and glucose delivery to the skeletal muscle cell for uptake.

## Post-Exercise Hemodynamics and Glucose Regulation in Individuals With Type 2 Diabetes

While the aforementioned studies were conducted on healthy, non-diabetic subjects, their findings highlight the importance of understanding the complex interactions amongst post-exercise hemodynamic and glucose regulation mechanisms in patients with T2D, who may engage in regular exercise to help manage their blood glucose levels. Sustained post-exercise vasodilation likely increases delivery of insulin to the microvasculature, where it has been shown to enhance capillary recruitment (Coggins et al., 2001; Vincent et al., 2004; Sjøberg et al., 2017) and may increase nutritive blood flow in patients with T2D (Clark, 2008), thus potentially circumventing insulin resistance in this population.

### Effects of Exercise on Glycemia in Patients With Type 2 Diabetes

It has been established that a single bout of dynamic exercise immediately confers beneficial post-exercise effects on glycemia in patients with T2D (Asano et al., 2014), thus serving as an effective strategy to help improve glycemic control in this population through repeated bouts of exercise (Way et al., 2016; Grace et al., 2017; Wake, 2020). The attenuation of hyperglycemia is primarily due to the enhanced uptake of glucose from the circulation, as shown in studies utilizing stable isotope tracers to assess glucose flux in patients with T2D (Borghouts et al., 2002; Boon et al., 2007). In this context, Boon et al. (2007) reported a significant decline in plasma glucose concentration in long-term-diagnosed T2D patients following 60 min of cycling exercise at 50% of W_max_. Comparisons in isotopic enrichments found that even though the plasma glucose rate of appearance (Ra) remained higher in T2D at rest and during post-exercise recovery, a significantly greater percentage of plasma glucose Ra was taken up and disappeared during and following exercise in the T2D patients compared to normoglycemic controls. Similarly, Borghouts et al. (2002) found that compared to non-diabetic control subjects, patients with T2D had greater reliance on plasma glucose oxidation vs. muscle glycogen oxidation for energy expenditure during 60 min of cycling exercise at 40% of VO_2peak_. These results support the notion that the exercise-induced decline in blood glucose concentration in patients with T2D is attributed to an acute increase in glucose uptake.

### Effects of Exercise on Endothelial Function in Patients With Type 2 Diabetes

Individuals with T2D often express early signs of cardiovascular co-morbidities, including endothelial dysfunction and arterial stiffness (Guerci et al., 2001; Frontoni et al., 2005), which may interfere with exercise-induced vasodilation following exercise. To determine whether exercise improves vasodilatory function in patients with T2D, Simões et al. (2013) compared post-exercise responses in patients with T2D to non-diabetics during a 20-min constant load exercise corresponding to 90% of LT, followed by a 45-min recovery period. The non-diabetic group showed slightly higher plasma kallikrein activity, bradykinin concentration, and nitric oxide (NO) concentration after exercise, all indicative of endothelium-dependent vasodilatory responses. To examine whether the release of these vasodilator substances is associated with the occurrence of PEH, the authors further reported that both groups did experience PEH, although it was more pronounced in the non-diabetic group compared to their counterparts with T2D. Moreover, as discussed below, these findings suggest that the bioavailability of NO released during exercise may be dependent on the intensity of the exercise bout.

### Dose-Dependent Effects of Exercise in Patients With Type 2 Diabetes

Several studies have suggested, given that patients can sustain it, higher intensity or longer duration exercise confers greater benefits to T2D management (Lima et al., 2008; Pellinger et al., 2017; Liu et al., 2019; Mendes et al., 2019; de Mello et al., 2021). The occurrence of PEH in T2D patients has been observed in a dose-dependent manner, such that a significant reduction in systolic blood pressure (SBP) occurred following 20 min of exercise at 90% of anaerobic threshold (AT), whereas diastolic blood pressure and mean arterial pressure were also reduced when the exercise intensity reached 110% of AT (Lima et al., 2008). Similarly, the increased concentration of exercise-induced NO was greater and the reduction in SBP was more pronounced when an exercise bout consisting of 20 min of cycling was conducted at 120% of LT, compared to 80% of LT (Asano et al., 2013). A recent study by Mendes et al. (2019) compared treadmill walking protocols for T2D patients involving either moderate-intensity steady exercise for 30 min at 50% of heart rate reserve (HRR) or high-intensity interval exercise (five sets of 3-min bouts at 70% of HRR interspersed by 3-min bouts at 30% of HRR). Including warm-up and cool-down, both protocols consisted of 40 min of exercise, followed by a 50-min recovery period. Acute effects during and following exercise showed that the high-intensity interval exercise reduced blood glucose to a greater extent compared to the moderate-intensity steady exercise.

Recent findings by Pellinger et al. (2017) suggest that in addition to intensity, exercise duration may also affect acute post-exercise femoral blood flow and vascular conductance in patients with T2D in a dose-dependent manner. In this study, individuals with well-controlled T2D participated in four different combinations of cycling exercise: 30 min at 40% VO_2peak_, 30 min at 60% VO_2peak_, 60 min at 40% VO_2peak_, and 60 min at 60% VO_2peak_. Sustained post-exercise hyperemia and reductions in SBP were observed in the latter three exercise protocols, suggesting that exercise must be at least moderate in intensity and/or prolonged in duration to evoke these acute hemodynamic responses in patients with T2D. Taken together, these findings suggest that higher intensity and/or longer duration exercise may promote PEH and improved glucose regulation in patients with T2D, at least in part due to enhanced post-exercise skeletal muscle blood flow.

## Discussion: Future Directions

Additional research is needed to further elucidate the complex relationship between PEH, post-exercise skeletal muscle blood flow and glucose regulation. Although the aforementioned research examining the potential relationship between post-exercise vasodilation and glucose regulation is compelling, most of the data derived from these investigations are associative. Therefore, more studies employing experimental manipulations designed to determine if there is a cause and effect relationship between these post-exercise phenomena are necessary.

Much of the research on healthy individuals may be extended to patients with T2D. For example, it is unclear if the histamine receptor-mediated post-exercise vasodilation that appears to sub-serve glucose regulation in healthy subjects (Pellinger et al., 2010, 2013; Emhoff et al., 2011) has the same effect in patients with T2D. In addition, the ability to estimate skeletal muscle membrane permeability (McConell et al., 2020) will allow investigations designed to better understand the interactions between glucose delivery, transport, and metabolism, in both healthy individuals and those with T2D.

In addition to investigations examining the acute effects of post-exercise vasodilation on glucose regulation, future investigations are needed to examine emerging interactions between vascular and metabolic adaptions to exercise. Along those lines, Van der Stede et al. (2021) observed that histamine receptor blockade blunted post-exercise muscle perfusion and increases in whole body insulin sensitivity in response to 6 weeks of high-intensity interval training in healthy males. Importantly, they also found that several histamine receptor-mediated adaptations were interrelated, as increases in VO_2m*ax*_ were related to changes in vascular function and whole-body insulin sensitivity. Moreover, a correlation was found between changes in capillary-fiber ratio and whole-body insulin sensitivity (Van der Stede et al., 2021). These findings are consistent with recent research suggesting that the post-exercise activation of H_1_- and H_2_-receptors upregulate several related pathways, including those related to metabolism, endothelial and vascular function (Romero et al., 2016), thereby highlighting important histaminergic adaptations to exercise that potentially impact both post-exercise blood flow and metabolic regulation.

In conclusion, recent investigations suggest that the post-exercise vasodilation that is often observed with PEH may aid in glucose regulation, via increased macrovascular and microvascular perfusion. Additional research is needed to further elucidate the relationship between post-exercise hemodynamics and glucose regulation in humans.

## Author Contributions

TP and C-AE wrote and revised the manuscript. Both authors contributed to the article and approved the submitted version.

## Conflict of Interest

The authors declare that the research was conducted in the absence of any commercial or financial relationships that could be construed as a potential conflict of interest.

## Publisher’s Note

All claims expressed in this article are solely those of the authors and do not necessarily represent those of their affiliated organizations, or those of the publisher, the editors and the reviewers. Any product that may be evaluated in this article, or claim that may be made by its manufacturer, is not guaranteed or endorsed by the publisher.
